# Supplementary material for: Cellsnake: a user-friendly tool for single-cell RNA sequencing analysis
Source: Gigascience. 2023 Oct 27;12:giad091. doi: 10.1093/gigascience/giad091 (PMC10603768; doi:10.1093/gigascience/giad091)
Supplement: giad091_GIGA-D-23-00148_Revision_1 [file giad091_giga-d-23-00148_revision_1.pdf]

GigaScience

cellsnake: a user-friendly tool for single-cell RNA sequencing analysis

--Manuscript Draft--

**Manuscript Number:**	GIGA-D-23-00148R1
**Full Title:**	cellsnake: a user-friendly tool for single-cell RNA sequencing analysis
**Article Type:**	Research
**Funding Information:**	Norges Forskningsråd (315483) — Prof. Frode Lars Jahnsen
**Abstract:**	Background<br><br>Single-cell RNA sequencing (scRNA-seq) provides high-resolution transcriptome data to understand the heterogeneity of cell populations at the single-cell level. Analysis of scRNA-seq data requires utilization of numerous computational tools. However, non-expert users usually experience installation issues, lack of critical functionality or batch analysis modes, and the steep learning curves of existing pipelines.<br><br>Results<br><br>We have developed cellsnake, a comprehensive, reproducible and accessible single-cell data analysis workflow, to overcome these problems. Cellsnake offers advanced features for standard users and facilitates downstream analyses in both R and Python environments. It is also designed for easy integration into existing workflows, allowing for rapid analyses of multiple samples.<br><br>Conclusion<br><br>As an open-source tool, cellsnake is accessible through Bioconda, PyPi, Docker and GitHub, making it a cost-effective and user-friendly option for researchers. By using cellsnake, researchers can streamline the analysis of scRNA-seq data and gain insights into the complex biology of single cells.
**Corresponding Author:**	Sinan U. Umu<br>University of Oslo<br>Oslo, Oslo NORWAY
**Corresponding Author Secondary Information:**	
**Corresponding Author's Institution:**	University of Oslo
**Corresponding Author's Secondary Institution:**	
**First Author:**	Sinan U. Umu
**First Author Secondary Information:**	
**Order of Authors:**	Sinan U. Umu
	Karoline Rapp Vander-Elst
	Victoria T. Karlsen
	Manto Chouliara
	Espen Sønderaal Bækkevold
	Frode Lars Jahnsen
	Diana Domanska
**Order of Authors Secondary Information:**	
**Response to Reviewers:**	We thank the reviewers for their valuable comments. We addressed all the issues. For this review, we also updated the cellsnake to a new version NUMBER. We fixed bugs

Powered by Editorial Manager® and ProduXion Manager® from Aries Systems Corporation

and improved performance. We rerun the submission version of cellsnake (v0.2.0.11) and updated the manuscript figures and repository files accordingly.

Reviewer reports:
Reviewer #1: The manuscript describes Cellsnake, a user-friendly tool for single-cell RNA sequencing analysis that targets non-expert users in the field of bioinformatics. Cellsnake operates as a command-line application, providing offline analysis capabilities for sensitive data. The integration of popular single-cell RNA-seq analysis software within Cellsnake, as described in Table 1, enhanced its utility as a comprehensive workflow. Cellsnake has different execution options (minimal, standard, and advanced) with varying outputs and execution times. The authors have provided well-structured online documentation, including helpful quick-start examples that facilitated easy understanding and usage of Cellsnake. The tool was tested using the Docker appliance and the provided fetal brain dataset and performed as expected. The manuscript explains the functions well, with the results reproduced from existing research using publicly available datasets.

The following issues need to be addressed by the authors.

1. The authors should include the citation for the Snakemake paper to acknowledge its contribution. https://doi.org/10.1093/bioinformatics/bts480

Thank you for the reviewer’s comment. We have added the Snakemake citation. We have updated the information in the section “Cellsnake workflow and tools”.

2. To support the claim of unique features in Cellsnake, a comparison with other similar methods, such as that on Galaxy (https://doi.org/10.1093/gigascience/giaa102), should be included.

Thank you for the reviewer’s comment. We added to the manuscript a new table (Table 2) in the Discussion section comparing the main features of three holistic analysis tools: cellsnake, Cellenics and single-cell omics workbench (i.e.Galaxy workflow).

3. It is recommended to host the Docker container image on both the GitHub Container Registry and the Docker Hub for better availability and redundancy. The authors should publish the Dockerfile to enable users to build a container image, if needed.

Thank you for the reviewer’s comment. Our Dockerfile is now available in our main repository (https://github.com/sinanugur/cellsnake/Dockerfile) and our workflow repository (https://github.com/sinanugur/scrna-workflow/Dockerfile). We added the publication version of Dockerfile to our Zenodo repository as well. Our Docker image is hosted at DockerHub (https://hub.docker.com/r/sinanugur/cellsnake).

4. Online documentation is missing a link to the fetal-liver example dataset (https://cellsnake.readthedocs.io/en/latest/fetalliver.html), which needs to be addressed. The fetal-brain dataset shared via Dropbox should also be deposited in the Zenodo repository to improve accessibility and long-term preservation.
Thank you for the reviewer’s comment. We included the Zenodo link of the dataset. We also updated our online documentation to cover all features of Cellsnake including how fetal-liver samples were processed.

5. To assist users who want to use Cellsnake as a Snakemake workflow, the tool documentation should provide clear instructions on how to run Cellsnake as a single snakemake pipeline. This would be useful for users who utilize existing workflow platforms to accept snakemake requests.
Thank you for the reviewer’s comment. We now updated the snakemake repository as well and put more comprehensive instructions (https://cellsnake.readthedocs.io/en/latest/). Cellsnake can be run as a tool or snakemake workflow as promised.

6. The benchmarking of Cellsnake must provide more precise specifications than simply referring to "a standard laptop" for computing requirements. My trial of

"cellsnake integrated standard" with the fetal-brain dataset took more than 17 h via Docker execution on my M1 Max MacBook Pro. This may be because the provided Docker image is AMD-based, which let my MacBook run the container on a VM, but the recommended computational specifications will help users. The GitHub issue of the Cellsnake repository also mentioned that the software is not tested on Windows Conda, which should be mentioned at least in the online documentation.

Thanks for the reviewer's comprehensive testing and commenting on the issue.

Unfortunately it is not possible to run cellsnake natively on Apple Silicon (M1 or newer) for now. For example, Kraken2 is not compatible with this architecture and our Docker image is therefore AMD64-based. It is however possible to run without a Docker container using the Bioconda package. Yet still it has to be forced to use Osx64 architecture and all the dependencies can be resolved. This offers a faster experience than Docker container for Apple laptops.

We noted this explicitly in our online documentation. As soon as there is a native solution, we will recreate a new Docker image.

7. In the Data Availability section, please ensure that the correct formatting and consistent identifiers are used for public data, such as replacing SRP129388 with PRJNA429950 and E-MTAB-7407 with PRJEB34784, specifying that these IDs are from the Bioproject database. It is important to mention that EGA files are under controlled access, requiring user permission for retrieval.

Thank you for the reviewer’s comment. We updated accordingly.

8. The references in the manuscript need to be properly formatted to ensure the inclusion of publication years and DOIs where available.
Thank you for the reviewer’s comment. Now all the references have DOI numbers and publication years.

9. The help message from the Cellsnake command indicates that its default values are set for human samples. The authors should mention in the manuscript that the pipeline is configured for human samples and requires further configuration for use with samples from other organisms. A step-by-step guide to configuring the setting for the other species, including the reference data download, would be helpful in obtaining more audiences.

Thank you for the reviewer’s comment. We now put a few examples and config files for other species. Our updated documentation can be accessed here (https://cellsnake.readthedocs.io/en/latest/)

Reviewer #2: This paper offers an open-source tool, i.e., cellsnake, to perform single-cell data analysis. This cellsnake tool offers advanced features for standard users and facilitates downstream analyses in both R and Python environments. It is also designed for easy integration into existing workflows, allowing for rapid analyses of multiple samples. I like the incorporation design of the metagenome analysis in this tool, which makes it different with other available tools in single-cell analysis.

1) I looked through their tutorial, and have a specific question regarding the resolution parameter. I wonder if this resolution argument needs to be pre-selected? Or the cellsnake tool can automatically select a resolution parameter?
Thank you for the reviewer’s comment. The resolution parameter is quite important since it selects granularity and many analyses on the downstream analyses mainly depend on how the clustering was done. Cellsnake can auto-select the resolution parameter and it works quite well if there are enough resources (memory and CPU). The user can also use the “clustree” plot to infer the optimum resolution parameter which is computationally less exhaustive.

2) Is it possible to add color legends in the umap? Rather than label all cell types on

Powered by Editorial Manager® and ProduXion Manager® from Aries Systems Corporation

	the umap. It can be very hard to distinguish the cell types, especially when there are many cell types available.<br>Thank you for the reviewer’s comment. This feature is already part of the cellsnake. In order to prepare the figures for the publication, we dropped legends. However, the legends are visible in our documentation and also in the supplementary files.<br><br>In large datasets, legends can cause overflow on the plots. We optimized the size of the plotting legends to prevent overflowing. Since the outputs are PDFs. The users can manually do some final work to create figure plots for their publication. We explore new ways to make cellsnake plots as easy as possible to use directly.<br><br>3) If the single-cell data is profiled from human tissue, is it also possible to use cellsnake to perform microbiome analysis?<br>Thank you for the reviewer’s comment. As long as the data is a single cell RNA dataset, microbiome analysis can be performed. It does not matter whether organisms or the tissue type. The only other requirement is the selection of the proper database to be able to use Kraken2.<br><br>4) I recommend the authors to compare cellsnake with other existing tools. Pros and cons need to be highlighted.<br>Thank you for the reviewer’s comment. As pointed out by the two reviewers, we included a table that compares the main features/functions of cellsnake with existing holistic tools/workflows. The table is added as table 3 in the discussion section.
**Additional Information:**	
**Question**	**Response**
Are you submitting this manuscript to a special series or article collection?	No
**Experimental design and statistics**<br><br><br>Full details of the experimental design and statistical methods used should be given in the Methods section, as detailed in our Minimum Standards Reporting Checklist. Information essential to interpreting the data presented should be made available in the figure legends.<br><br><br>Have you included all the information requested in your manuscript?	Yes
**Resources**<br><br><br>A description of all resources used, including antibodies, cell lines, animals and software tools, with enough information to allow them to be uniquely identified, should be included in the Methods section. Authors are strongly encouraged to cite Research Resource Identifiers (RRIDs) for antibodies, model organisms and tools, where possible.	Yes

Powered by Editorial Manager® and ProduXion Manager® from Aries Systems Corporation

Have you included the information requested as detailed in our Minimum Standards Reporting Checklist?	
**Availability of data and materials**<br><br>All datasets and code on which the conclusions of the paper rely must be either included in your submission or deposited in publicly available repositories (where available and ethically appropriate), referencing such data using a unique identifier in the references and in the "Availability of Data and Materials" section of your manuscript.<br><br>Have you have met the above requirement as detailed in our Minimum Standards Reporting Checklist?	Yes

Click here to access/download;Manuscript;cellsnake manuscript revised.docx

cellsnake: a user-friendly tool for single-cell RNA sequencing analysis

Sinan U. Umu[1,*], Karoline Rapp Vander-Elst[2], Victoria T. Karlsen[2], Manto Chouliara[2], Espen Sønderaal Bækkevold[2,3], Frode Lars Jahnsen[1,2] and Diana Domanska[2,4]

1 Department of Pathology, Institute of Clinical Medicine, University of Oslo, Oslo, Norway

2 Department of Pathology, Oslo University Hospital-Rikshospitalet, Oslo, Norway

3 Institute of Oral Biology, University of Oslo, Oslo, Norway

4 Department of Microbiology, University of Oslo, Rikshospitalet, Oslo, Norway

*corresponding author: sinanuu@uio.no

Abstract

Background

Single-cell RNA sequencing (scRNA-seq) provides high-resolution transcriptome data to understand the heterogeneity of cell populations at the single-cell level. The analysis of scRNA-seq data requires the utilization of numerous computational tools. However, non-expert users usually experience installation issues, a lack of critical functionality or batch analysis modes, and the steep learning curves of existing pipelines.

Results

We have developed cellsnake, a comprehensive, reproducible, and accessible single-cell data analysis workflow, to overcome these problems. Cellsnake offers advanced features for standard users and facilitates downstream analyses in both R and Python environments. It is also designed for easy integration into existing workflows, allowing for rapid analyses of multiple samples.

Conclusion

As an open-source tool, cellsnake is accessible through Bioconda, PyPi, Docker, and GitHub, making it a cost-effective and user-friendly option for researchers. By using cellsnake, researchers can streamline the analysis of scRNA-seq data and gain insights into the complex biology of single cells.

Background

Single-cell RNA sequencing (scRNA-seq) is a method used to study gene expression at the single-cell level. This stands in contrast to bulk RNA sequencing, which provides information only on the average transcript expression within a population of cells. With recent technological advancements and decreasing sequencing costs, scRNA-seq has become increasingly accessible, enabling researchers to identify novel cell types, cell states, and cellular interactions [1–4].

A standard scRNA-seq bioinformatics workflow typically involves several steps, including data filtering, normalization, scaling, dimensionality reduction, clustering, visualization, differential expression analysis, functional analysis, and annotation [4,5]. Various analysis workflows for different platforms (i.e. 10x Genomics, Drop-seq, inDrops, SMART-seq2, and Fluidigm C1) have been developed to process, analyze and holistically visualize scRNA-seq data [2,6–8]. Popular workflows like Seurat [9], SingleCellExperiment (of Bioconductor) [7], and Scanpy [6] have extensive features for scRNA analysis. The analysis of scRNA-seq data poses several challenges, including the high-dimensional data structure, technical issues (e.g. dead cells, doublets, and low unique molecular identifier (UMI) counts), batch effects, low expression levels, and the presence of complex cell subsets with multiple cell states [5]. To address these, a variety of supplementary bioinformatics tools have been developed. While some of these can be integrated into existing workflows, many require substantial expertise and bioinformatics knowledge.

Another challenge is working with multiple scRNA-seq datasets. Comprehensive documentation for the analysis of a single sample using recommended parameters is usually provided. However, it is hard for a regular user to keep track of all the decisions taken during analyses, especially if more than one sample is available. This also creates challenges if one wants to see the effect of basic parameter changes and document the results for further

hypothesis testing. It is also challenging to harness the power of high-performance computing (HPC) systems when needed. There are some efforts to make batch analysis, such as the cloud-based system SingleCAnalyzer [10], the R package scTyper [11], the web application Cellenics (open-source software of Biomage), and Single-Cell Omics workbench on Galaxy (singlecell.usegalaxy.eu). Cellranger from 10x genomics also provides dataset clustering and basic differential expression analysis [12] for initial quality control (QC). However, all these workflows have limited functionality or were designed for a specific need. Online (or cluster-based) solutions might also not be suitable due to data privacy rules for sensitive data or do not provide compatible files (e.g. R data files) for downstream analysis on another platform.

Here, we introduce cellsnake, a platform-independent command-line application and pipeline for scRNA-seq analysis. Cellsnake provides a reproducible, flexible, and accessible solution for most scRNA-seq data analysis applications. One of the key features of cellsnake is its ability to utilize different scRNA-seq algorithms to simplify tasks such as automatic mitochondrial (MT) gene trimming, selection of optimal clustering resolution, doublet filtering, visualization of marker genes, enrichment analysis, and pathway analysis. Cellsnake also allows parallelization and readily utilizes high-performance computing (HPC) platforms. In addition to that, cellsnake provides metagenome analysis if unmapped reads are available. Another advantage of cellsnake is its ability to generate intermediate files (such as R data files) that can be stored, extracted, shared, or used later for more advanced analyses or for reproducibility purposes. With cellsnake, researchers can perform scRNA-seq data analysis in a reproducible and efficient manner, without requiring extensive bioinformatics expertise.

Methods

Cellsnake workflow and tools

The cellsnake wrapper was written in Python, while the main workflow was implemented in Snakemake [13]. To find optimal cluster resolution, we utilized clustree [14]. Seurat analysis pipeline [8] provides all the main functions required for processing scRNA data in cellsnake. These functions are wrapped into different R scripts which can also be used as standalone scripts by advanced users. Cellsnake facilitates automatic format conversion when required. For instance, CellTypist [15] requires AnnData format, and the workflow converts the files back to the required file format in R. By default, cellsnake stores files into two folders: analyses and results. The analyses folder contains metadata and R data files, which can be accessed by the user. Seurat is used for integration, and after integration, the workflow runs on the integrated dataset automatically, and the output files are stored in separate folders (i.e. analyses_integrated and results_integrated).

Parameter selection and autodetection

Cellsnake provides Seurat's default values for fundamental parameters like min.cells (i.e features detected at least this many cells) or min.features (i.e. cells at least this many features). In addition, non-default parameters can be provided using a YAML file, and a YAML file template can be printed and edited. Cellsnake determines which principal component exhibits cumulative percent greater than 90% and % variation associated with the principal component as less than 5 (as described hbctraining.github.io/scRNA-seq/lessons/elbow_plot_metric.html). To filter MT genes, cellsnake uses the miQC tool [16]. If that fails, it uses the median absolute deviation of the MT gene expression as an alternative. MultiK algorithm [17] is used to determine optimal resolution detection and doublet filtering is done using the DoubletFinder tool [18]. Autodetection of parameters is not offered as a default option in cellsnake due to its computational expense and potential for failure with large sample sizes. Cellsnake utilizes a special directory structure for MT

percentage and resolution the results will be saved in different folders named after the selected parameters. These results are not overwritten and can be reviewed later, or the parameters can be modified for further investigation.

Cellsnake testing and benchmarks

To test cellsnake, we obtained four samples containing exclusively macrophages from gut mucosal tissue [19], along with two fetal brain datasets [20] and six fetal liver datasets [21]. The fetal brain datasets were provided in matrix file format, while the other datasets were in FASTQ format and were processed by Cellranger (v.7.0.0) with the default settings and the default databases. For a comprehensive evaluation, we compared the features of Cellsnake with two other holistic tools, Cellenics and Single Cell Omics workbench (https://singlecell.usegalaxy.eu/). The Cellenics community instance (https://scp.biomage.net/) is hosted by Biomage (https://biomage.net/).

Results

Cellsnake can be run either as a Snakemake workflow or as a standalone tool

Cellsnake utilizes a variety of tools and algorithms (Table 1) and consists of two primary components: the main workflow and the wrapper. The cellsnake wrapper assists with the main workflow and provides an easy-to-use option for users. The workflow (Fig. 1) is primarily designed using the Seurat pipeline (v4.2) and the Snakemake workflow manager. As needed, the workflow integrates various algorithms to enhance the basic functionality of Seurat. For instance, when one droplet encapsulates more than one cell, it appears as a single cell and can affect the downstream analysis. Addressing this issue in the workflow is crucial [22]. A distinctive feature of cellsnake is its default doublet filtering option, a functionality not included in the standard Seurat pipeline. Users can also adjust other parameters by modifying the configuration files, which are formatted in YAML. This flexibility empowers precise analysis of scRNA-seq data.

Cellsnake covers most of the methods offered by Seurat, including integration. The workflow is automatically repeated for an integrated dataset once the analyses have been concluded for all individual samples available in the study (i.e QC, filtering etc.). Analysis outcomes such as dimension reduction, clustering, differential expression analysis, functional enrichment, and cell type annotations are reported for the integrated sample. Since the datasets individually passed the initial QC and are trimmed for artifacts, these steps are skipped by the workflow. Cellsnake can also generate publication-ready plots for both individual and integrated samples. It also automatically produces plots for markers (i.e. genes) which can be investigated to better understand the predicted clusters (i.e. cell subsets). Additionally, cellsnake provides the option to produce supplementary plots, featuring dimension reduction and expression images for selected genes or markers. This functionality adds a valuable level of customization to the analysis, enabling the user to explore targeted genes or markers of interest in greater detail.

The input of cellsnake can be either Cellranger output directories for batch analysis or single expression matrix files (e.g. h5 files) for individual sample processing. Cellsnake automatically detects the input format and runs accordingly with minimal user intervention and with minimal lines of input commands (Table 2). The Cellsnake workflow offers three primary modes with distinct options: minimal, standard, and advanced. The minimal mode is suitable for fast analysis, parameter selection, and downstream integration. Fundamental parameters, such as filtering thresholds and clustering resolution can be determined via a minimal run at an early stage which will reduce computational cost. Standard and advanced workflow modes contain additional features and algorithms (Table 2).

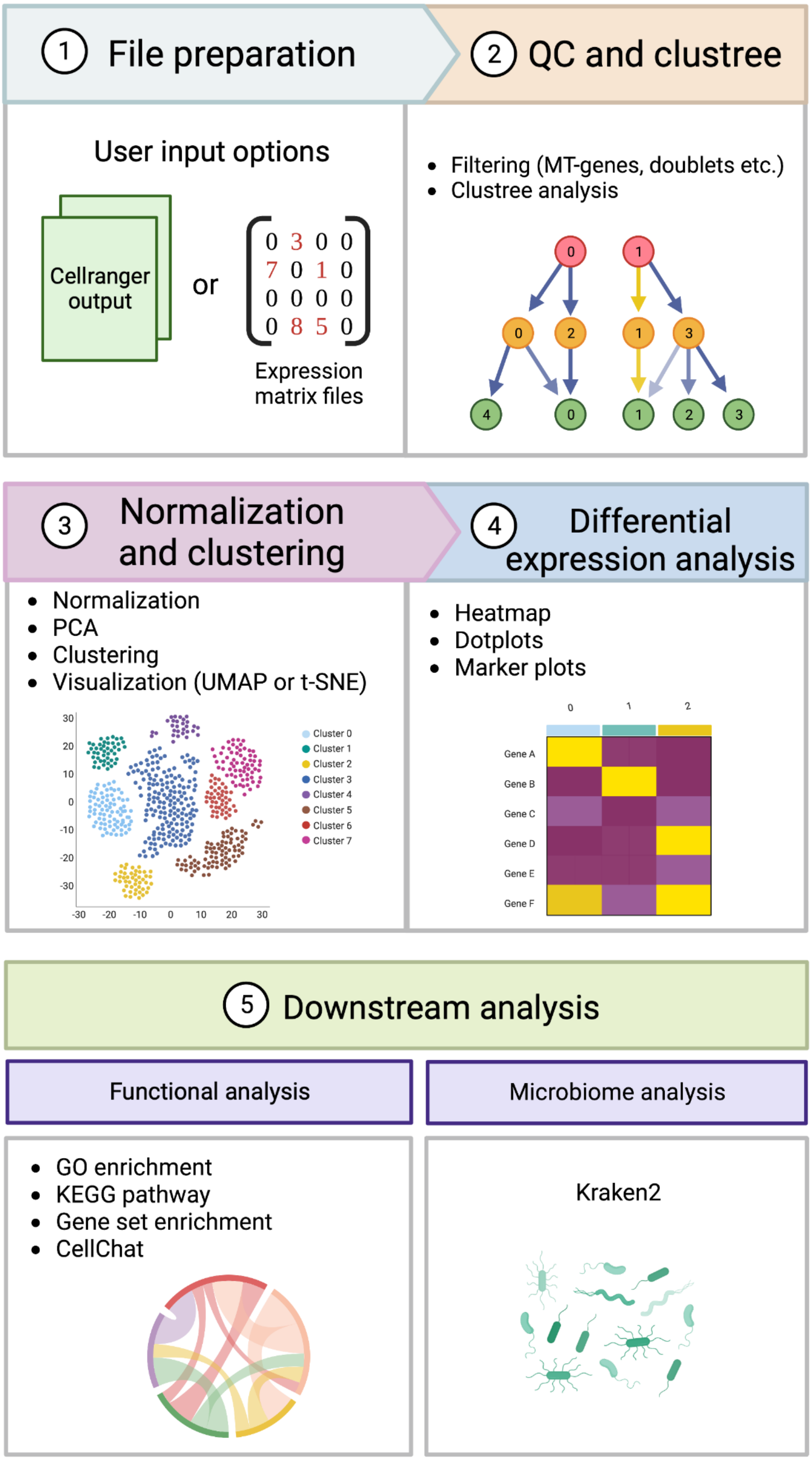

Figure 1. Overview of the scRNA-seq pipeline in cellsnake. (1) Cellsnake can accept the output files from Cellranger in addition to raw expression matrix files if provided in an appropriate format. (2) QC is performed by filtering out MT-genes, doublets, and cells with a low gene number as examples. Clustree is then used to find the optimal resolution for the dimensionality reduction. (3) Afterward, the dataset is normalized and scaled before the PCA

analysis and visualized by UMAP or tSNE. (4) To find the differences in gene expression levels within the dataset differential gene expression analysis is performed with several outputs such as heatmaps, dot plots, and marker plots. (5) To get an even better insight into the dataset, the pipeline contains several functional analyses such as GO enrichment, KEGG pathway, gene set enrichment, and CellChat. Metagenome analysis is also available if the input file from step 1 is the direct output from Cellranger. This is done by using the metagenomics tool Kraken2.

Table 1. An overview of the tools and algorithms used in the cellsnake workflow, as well as an explanation of what they do and which versions are used.

**Tool**	**Version**	**Reference**	**Notes**
Seurat	4.2.0	[8]	Main analysis platform
SeuratDisk	0.9020	github.com/mojaveazure/seurat-disk/	Format converter
Clustree	0.5.0	[14]	Clustering interrogation
MultiK	1.0	[17]	Optimal cluster detection
miQC	1.6.0	[16]	Auto MT gene trimming
DoubletFinder	2.0.3	[18]	Doublet detection
SingleR	2.0.0	[23]	Cell type annotation
CellTypist	1.2.0	[15]	Cell type annotation
Kraken2	2.1.2	[24]	Metagenomics
CellChat	1.6.1	[25]	Ligand-receptor analysis and miscellaneous
clusterProfiler	4.4.4	[26]	KEGG, GO and module

			enrichment
Monocle3	1.0.0	[27]	Cell trajectory and velocity

Table 2. Cellsnake commands and a summary of their outputs.

**Mode**	**Outputs**	**How to run?**
cellsnake minimal	Dimension reduction plots, QC metrics, technical plots (MT, counts, gene, feature), clustree plot	`$ cellsnake minimal data`<br>OR<br>`$ snakemake -j 5 --config option=minimal`
cellsnake standard	All minimal outputs and CellTypist, singleR annotations, enrichment analyses tables, trajectory plots, summarized marker plots	`$ cellsnake standard data`<br>OR<br>`$ snakemake -j 5 --config option=standard`
cellsnake advanced	All standard outputs and CellChat results, detailed top markers per cluster plots	`$ cellsnake advanced data`<br>OR<br>`$ snakemake -j 5 --config option=advanced`
cellsnake integrate	A single integrated object for analysis.	`$ cellsnake integrate data`<br>OR<br>`$ snakemake -j 5 --config option=integrate`

* data folder may contain multiple samples and this will trigger a batch analysis.

Reanalyses of publicly available datasets using cellsnake

We showcase some features of the pipeline using publicly available datasets. The first dataset is from the fetal brain containing (only) count tables from two samples (Fig. 2 and Fig. 3). We processed two samples using the default settings (e.g. MT filtering threshold 10

percent and resolution parameter 0.8). Minimal mode only takes four minutes in a laptop for two samples of the fetal brain dataset. Another five minutes is enough for both integration and processing of the integrated sample with minimal mode. The user can decide on the parameters early on (Fig. 2) and the standard mode will finish in 50 minutes without parallel processing. Cellsnake utilizes different tools and provides outputs for all as figures (supplementary figures 1-6) or as tables.

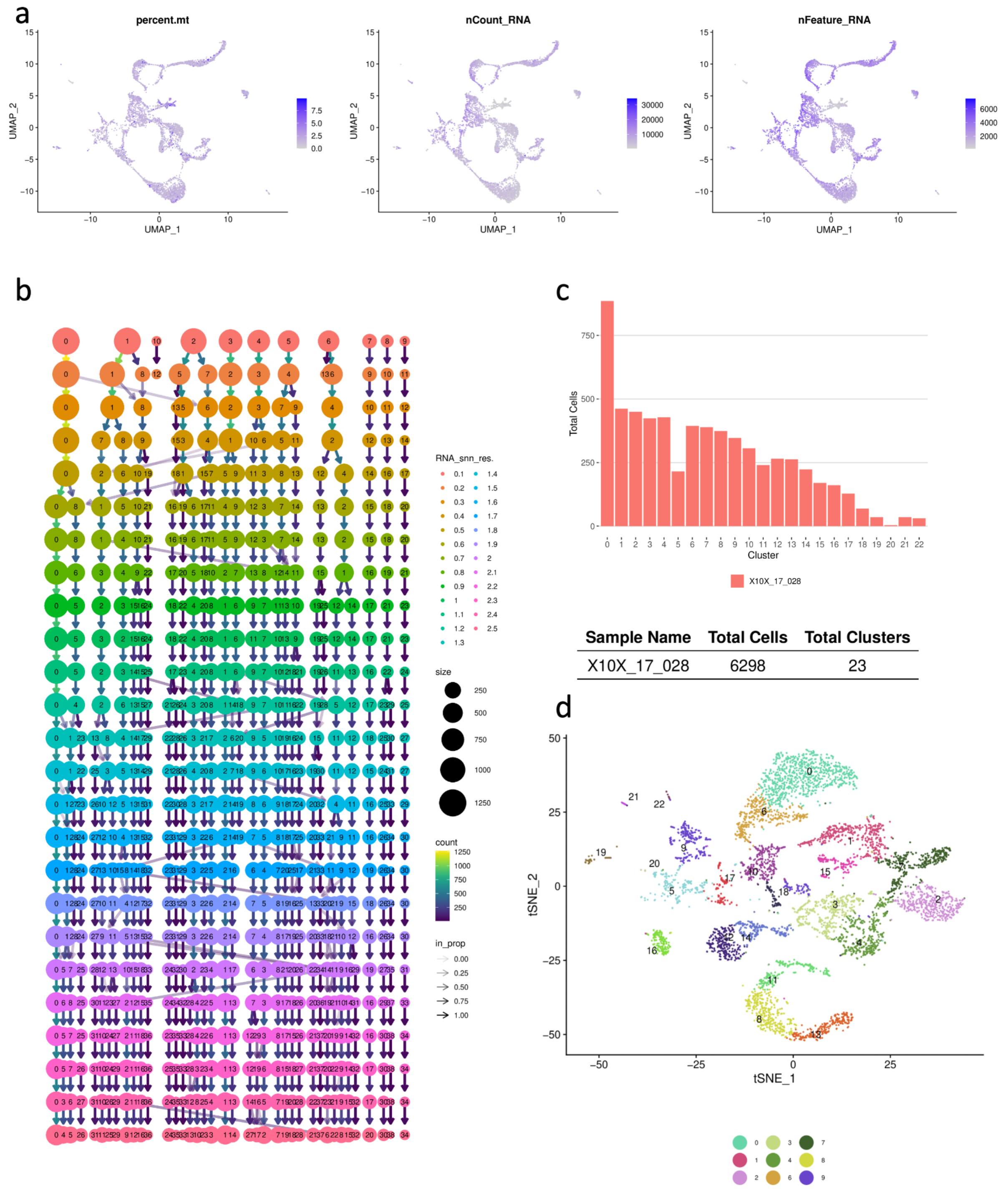

Figure 2. Cellsnake quickly generates standard output plots that include technical information. a) The user can investigate the fundamental statistics like MT gene percentage, number of genes detected, and reads mapped per cell information. Here the results shown are based on one of the fetal brain samples. b) Clustree analysis is not part of the Seurat pipeline but cellsnake offers this by default. This plot can be used to find the

optimal number of clusters. c) The selected resolution resulted in 23 clusters and 6298 cells passed the filtering thresholds (after filtering doublets and low-quality cells). d) tSNE plot shows the clusters. Cellsnake prints only the top clusters in the legend to prevent overplotting. The user will get UMAP, PCA, and tSNE plots by default.

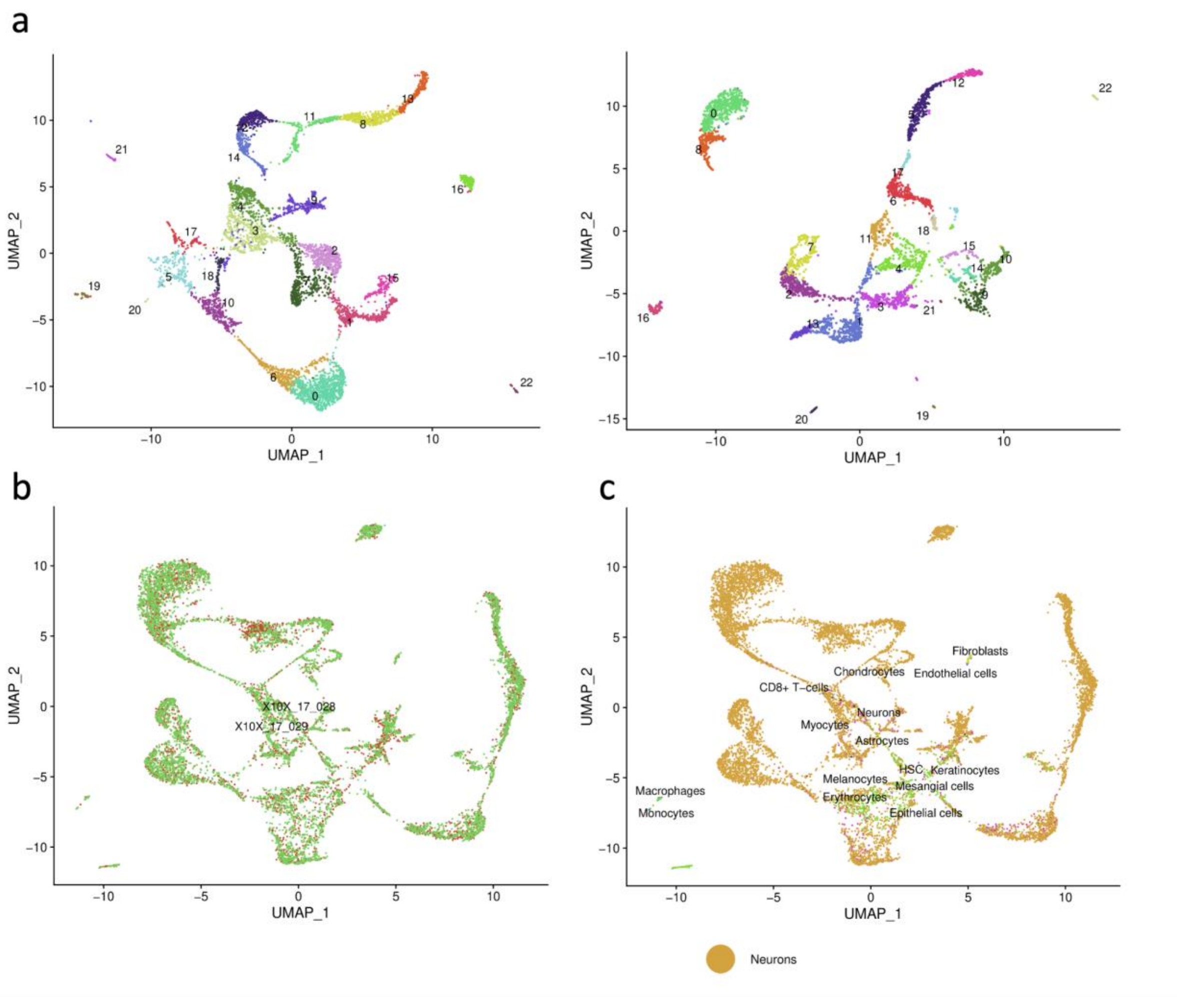

Figure 3. Cellsnake processes integrated samples similar to the individual samples and generates the same plots. a) The UMAP plots were generated for two samples from the fetal brain dataset, seen in the first and second panels. b) The UMAP plot shows clusters for the integrated samples. c) The UMAP plot shows cluster annotation based on the singleR package "BlueprintEncodeData" model predictions. The results showed the cells were mostly predicted as neurons which are consistent with the dataset but there are also some mispredictions. The detailed annotations can be accessed as Excel tables and heatmaps.

The second dataset is from the fetal liver containing three CD45+ and three CD45- FACS sorted samples from three different donors (Fig. 4A). This time we selected automatic filtering of MT gene abundant cells rather than a hard cut-off when pre-processing the samples. In total, 29045 cells passed the filtering threshold. The standard workflow took 3 hours with only two CPU cores on a standard laptop, which is enough for most use cases. The samples were later integrated and the optimal number of clusters was predicted automatically. The separation of two groups (Fig. 4A and 4B) in the integrated dataset is similar to what was reported in the original study [21], which indicates that cellsnake is capable of reproducing key findings from published studies. The differential expression analysis also reveals that the AHSP gene is highly expressed in CD45+ samples, which is in line with the known function of this gene in erythroid cells.

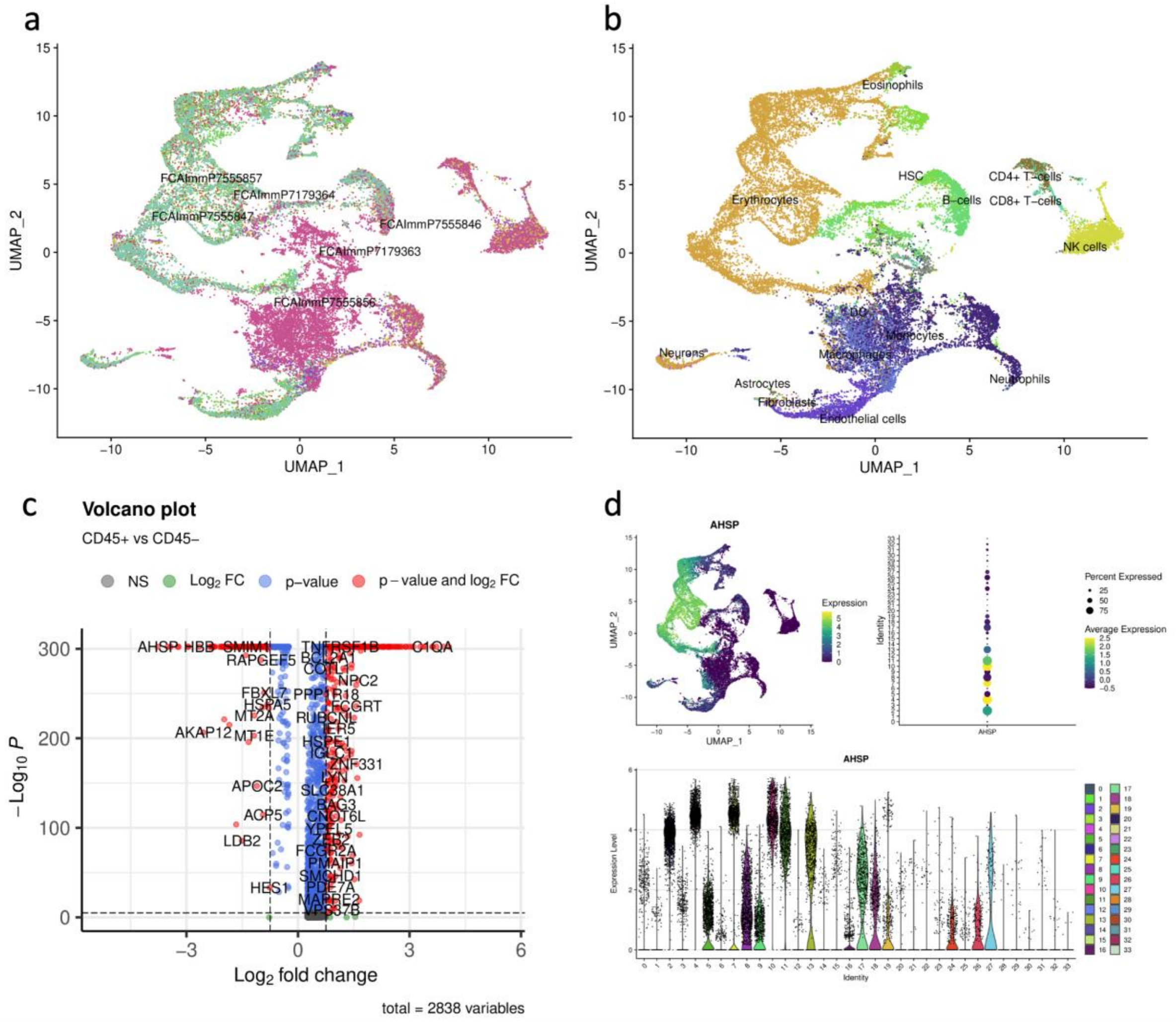

Figure 4. The fetal liver dataset consists of six FACS-sorted samples, integrated by cellsnake. a) Cellsnake displays integrated UMAP plot, and labels, and b) annotates the clusters. c) The user can provide the clinical information which shows differentially expressed genes among two groups. d) It is also possible to visualize selected marker genes. For example, the AHSP gene is upregulated in CD45+ samples compared to CD45- samples.

Cellsnake can analyze metagenomics from single-cell data

Another unique feature of Cellsnake is its ability to perform metagenomics analysis using Kraken2. If a database is provided, Cellsnake will automatically run Kraken2. After collapsing read counts to a taxonomic level based on user input, such as genus or phylum, results are

reported accordingly. Cellsnake provides metagenomic results in the form of dimension reduction plots and barplots, and users can load metadata into R for personalized downstream analysis.

This feature was tested on four samples from mucosal macrophages, with automatic trimming of MT genes and selection of resolution (Fig. 5). Cellsnake reported results based on the optimal number of clusters, and non-human material detected by Kraken2 is visualized on integrated UMAP plots (Fig. 5a,b). Users can also obtain a detailed list of results based on the selected taxonomic level in an Excel file.

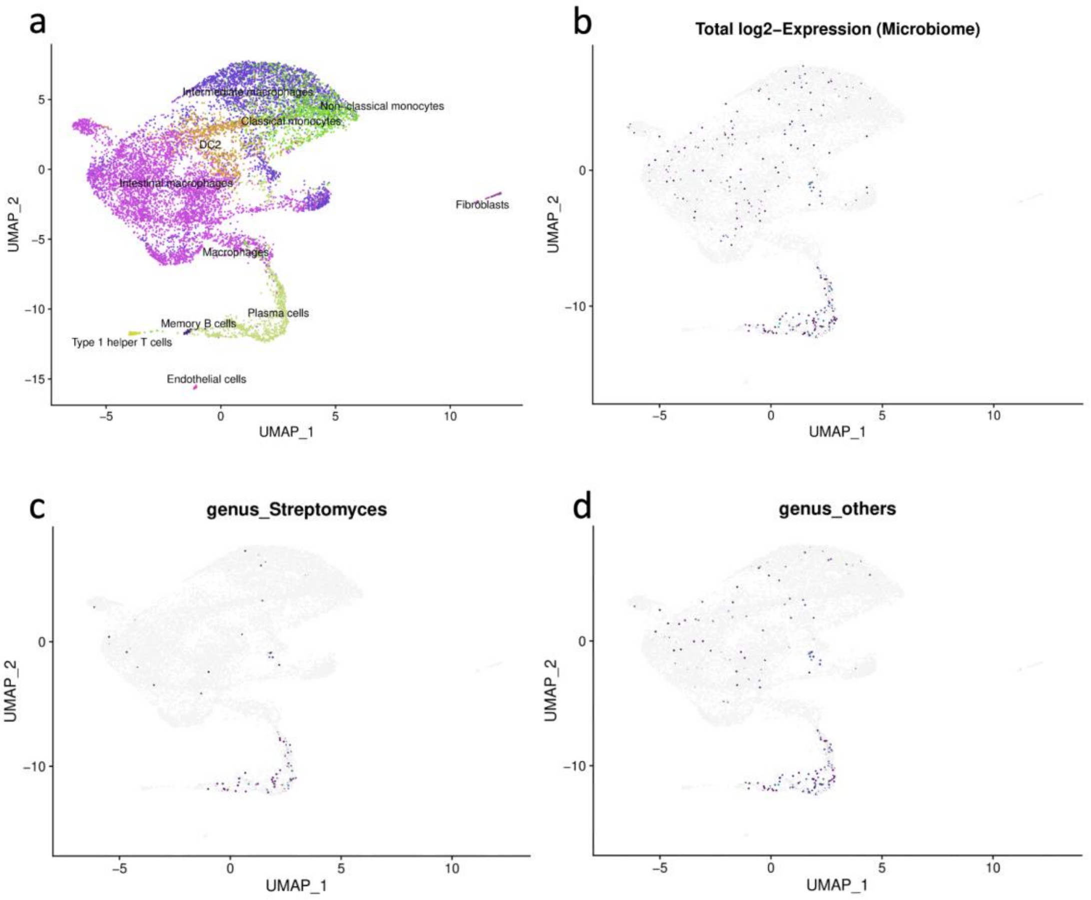

Figure 5. Cellsnake's metagenomics feature was tested on mucosal macrophages. a) Four samples were integrated. The clusters were predicted and annotated using the CellTypist immune model. b) The cells annotated as "plasma cells" contain the highest

number of bacterial reads. c) The foreign reads were mostly associated with Streptomyces. d) Cellsnake reports the top 10 most prevalent taxonomic groups by default. The rest collapsed and were reported as “others”. The user can select the desired taxonomic level (in this case, it was genus). All results are also saved as tables which include reads detected per cluster and annotation.

Discussion

In recent years, there has been an increasing interest in scRNA-seq as it is a powerful technique for understanding the cellular heterogeneity of tissues and organs. However, the scRNA-seq data analysis can be complex and time-consuming. Cellsnake was designed to simplify this process, enabling researchers without extensive bioinformatics experience to easily analyze their data. It includes a range of automated preprocessing and downstream analysis tools and also provides advanced features for additional analysis. Its user-friendly interface and reproducibility features make it a valuable tool for researchers seeking to understand transcriptional heterogeneity in tissues at single-cell resolution.

Cellsnake has several critical functionalities for scRNA-seq data analysis. It includes preprocessing steps such as QC, filtering, and parameter auto-selection, and also has downstream analysis tools for identifying differentially expressed genes, performing clustering, visualization, and exploring cell type-specific gene expression patterns. These features are crucial for characterizing cell subpopulations and identifying specific genes and pathways associated with them. Cellsnake also includes advanced features such as supporting the integration of multiple scRNA-seq datasets to identify shared and unique cell types across different tissues or conditions. Cellsnake also ensures reproducibility by creating separate folders when required, restricting the versions of the tools in the environment, saving config files with the cellsnake version, explicitly sharing different images

for each version in the Docker repository, and storing results for downstream analysis by default. In comparison to other tools (Table 3), cellsnake has several advantages, including a comprehensive range of tool utilization, unique features, the ability to run locally or on HPC platforms, and seamless integration with other workflows using Docker or Bioconda. Additionally, cellsnake also provides RDS files to enhance data sharing and accessibility.

Table 3. Standard features of cellsnake compared to available holistic tools/workflows.

	Cellsnake	Cellenics	Single Cell Omics Workbench
Platform	Snakemake/Python wrapper/Docker	Web based	Web based (Galaxy)
Input file type	Count tables (10X or others), R Data File	Count tables (10X)	Count tables (10X), FASTQ and others
Doublet filtering	Yes	Yes	No
MT gene filtering	Yes (auto)	Yes (auto)	Yes
Find clusters	Yes (auto)	Yes	Yes
Clustree plot	Yes	No	No
Differential expression analysis	Yes	Yes	Yes
Enrichment analysis	KEGG and GO	No	No
Celltype Annotation	Yes	Yes	No
Detailed gene expression plots	Yes	No	No
Metagenome analysis	Yes	No	No
Trajectory analysis	Yes	Yes	Yes

Integration	Yes (Seurat only)	Yes (various algorithms)	Yes
Output and downstream analysis	Plot files, expression tables, Seurat RDS files and excel files etc.	Plots and expression tables	Miscellaneous

Recent studies have shown that the heterogeneity in microbiota and the present cell types along with their functions are co-dependent [28]. Cell-associated microbial reads can be identified in scRNA-seq data [29]. Cellsnake uses Kraken2 [24] to analyze this data and cellsnake provides the ability to fine-tune parameters to increase sensitivity and/or specificity and to use personal databases. This can help researchers identify potential microbial associations with host cells and tissues. Some of these microbial hits can originate from environmental contamination or can be false positives. These outcomes might not necessarily reflect real biological associations; nevertheless, the results may provide valuable insights for QC such as recognizing potential contamination sources.

There are some limitations of the workflow that need to be addressed. Firstly, cellsnake requires disk space to keep track of the entire pipeline, including metadata files that are required for advanced downstream analysis. Although the users can delete large files, they may want to keep metadata files for reproducing the results at a later time. Secondly, the fully-featured workflow relies on Cellranger outputs from 10x Genomics platform, which may not always be available. Even though cellsnake was designed and tested utilizing this platform, it can still use the count matrix files from other platforms, such as the fetal brain dataset. Third, while cellsnake has moderate performance in terms of memory and speed on standard workstations for an average number of cells, the auto-detection of parameters

(e.g., resolution parameter) can be slow when processing samples with a large number of cells. To improve performance, a parallel version of the MultiK tool was used, which is not officially supported by the authors of MultiK (see materials and methods). Finally, the underlying tools utilized by cellsnake may involve various parameters. The fundamental parameters can be adjusted by the user and supplied through the configuration files, while the rest are set to default values. This approach was preferred to make the workflow more user-friendly.

In conclusion, cellsnake is a convenient and adaptable tool, empowering researchers to analyze scRNA-seq data in a reproducible and customizable manner. With its advanced features and streamlined workflow, cellsnake stands as a valuable bioinformatics asset for investigating cellular heterogeneity and gene expression patterns at single-cell resolution within tissues.

Future Directions

Accurate bioinformatics software requires long-term development and commitment to the project [30]. It is also a major problem in the field that many projects are abandoned after publication, becoming unusable and outdated. For instance, cerebroApp [31], a component of cellsnake's development version, was dropped as it is no longer in active development. Cellsnake is an open-source tool that is actively developed, allowing anyone to open pull requests and report issues on its GitHub page. To keep the software bug-free and streamlined, future developments of cellsnake will involve incorporating new tools, such as the latest Seurat version, and removing obsolete tools from the main workflow. The users can access the previous releases for reproducibility. Although cellsnake is mainly designed for the 10X Genomics single-cell platform, we plan to expand its compatibility with other platforms and offer additional support for various input formats. Our aim is for cellsnake to

become an essential toolkit for fast, accurate, tunable, and comprehensive scRNA data analysis.

Data availability

The publicly available datasets for the fetal brain and liver are available under accessions PRJNA429950 and PRJEB34784, respectively. Macrophage-only samples from gut mucosal tissue are deposited in the European Genome-Phenome Archive (EGA) under the following accession numbers: EGAD00001007765 and EGAS00001005377. The EGA deposited files are under controlled access, requiring the data access committee permission for retrieval. The cellsnake analysis results on test samples are available at https://doi.org/10.5281/zenodo.8282676. A copy of the fetal brain dataset can also be found in our frozen Zenodo repository.

Availability and Requirements

- Project name: cellsnake
- Project homepage: https://github.com/sinanugur/cellsnake
- Documentation: https://cellsnake.readthedocs.io/en/latest/
- RRID: SCR_023666
- Operating system: Platform independent
- Programming language: Python, R
- Other requirements: Python 3.8 or higher, R 4.2.2
- License: MIT
- PyPi: https://pypi.org/project/cellsnake
- Bioconda: https://anaconda.org/bioconda/cellsnake
- Docker: https://hub.docker.com/r/sinanugur/cellsnake
- Snakemake workflow: https://github.com/sinanugur/scrna-workflow

Author contributions

SUU devised the project, created the workflow, wrapper, and R scripts, and drafted the manuscript with input from all authors. KRV contributed to the figures and the R scripts. VTK contributed to the R scripts. MC contributed to the R scripts and revised the preliminary manuscript. ESB revised the manuscript and acquired financial support. FLJ revised the manuscript and acquired financial support. DD supervised the project, contributed to the R scripts, revised the manuscript, and acquired financial support.

Funding

This work was supported by The Research Council of Norway (project number 315483).

Competing interests

None declared.

References

1. Saliba A-E, Westermann AJ, Gorski SA, Vogel J. Single-cell RNA-seq: advances and future challenges. *Nucleic Acids Res*. 2014; doi: 10.1093/nar/gku555.

2. Jovic D, Liang X, Zeng H, Lin L, Xu F, Luo Y. Single-cell RNA sequencing technologies and applications: A brief overview. *Clin Transl Med*. Wiley; 2022; doi: 10.1002/ctm2.694.

3. Bacher R, Kendziorski C. Design and computational analysis of single-cell RNA-sequencing experiments. *Genome Biol*. 2016; doi: 10.1186/s13059-016-0927-y.

4. Nayak R, Hasija Y. A hitchhiker's guide to single-cell transcriptomics and data analysis pipelines. *Genomics*. 2021; doi: 10.1016/j.ygeno.2021.01.007.

5. Lähnemann D, Köster J, Szczurek E, McCarthy DJ, Hicks SC, Robinson MD, et al.. Eleven grand challenges in single-cell data science. *Genome Biol*. 2020; doi: 10.1186/s13059-020-1926-6.

6. Wolf FA, Angerer P, Theis FJ. SCANPY: large-scale single-cell gene expression data analysis. *Genome Biol*. 2018; doi: 10.1186/s13059-017-1382-0.

7. Amezquita RA, Lun ATL, Becht E, Carey VJ, Carpp LN, Geistlinger L, et al.. Orchestrating single-cell analysis with Bioconductor. *Nat Methods*. 2020; doi: 10.1038/s41592-019-0654-x.

8. Hao Y, Hao S, Andersen-Nissen E, Mauck WM 3rd, Zheng S, Butler A, et al.. Integrated analysis of multimodal single-cell data. *Cell*. 2021; doi: 10.1016/j.cell.2021.04.048.

9. Satija R, Farrell JA, Gennert D, Schier AF, Regev A. Spatial reconstruction of single-cell gene expression data. *Nat Biotechnol*. 2015; doi: 10.1038/nbt.3192.

10. Prieto C, Barrios D, Villaverde A. SingleCAnalyzer: Interactive Analysis of Single Cell RNA-Seq Data on the Cloud. *Front Bioinform*. 2022; doi: 10.3389/fbinf.2022.793309.

11. Choi J-H, In Kim H, Woo HG. scTyper: a comprehensive pipeline for the cell typing analysis of single-cell RNA-seq data. *BMC Bioinformatics*. 2020; doi: 10.1186/s12859-020-03700-5.

12. Zheng GXY, Terry JM, Belgrader P, Ryvkin P, Bent ZW, Wilson R, et al.. Massively parallel digital transcriptional profiling of single cells. *Nat Commun*. 2017; doi: 10.1038/ncomms14049.

13. Mölder F, Jablonski KP, Letcher B, Hall MB, Tomkins-Tinch CH, Sochat V, et al.. Sustainable data analysis with Snakemake. *F1000Res*. 2021; doi: 10.12688/f1000research.29032.2.

14. Zappia L, Oshlack A. Clustering trees: a visualization for evaluating clusterings at multiple resolutions. *Gigascience*. 2018; doi: 10.1093/gigascience/giy083.

15. Domínguez Conde C, Xu C, Jarvis LB, Rainbow DB, Wells SB, Gomes T, et al.. Cross-tissue immune cell analysis reveals tissue-specific features in humans. *Science*. 2022; doi: 10.1126/science.abl5197.

16. Hippen AA, Falco MM, Weber LM, Erkan EP, Zhang K, Doherty JA, et al.. miQC: An adaptive probabilistic framework for quality control of single-cell RNA-sequencing data. *PLoS Comput Biol*. 2021; doi: 10.1371/journal.pcbi.1009290.

17. Liu S, Thennavan A, Garay JP, Marron JS, Perou CM. MultiK: an automated tool to determine optimal cluster numbers in single-cell RNA sequencing data. *Genome Biol*. 2021; doi: 10.1186/s13059-021-02445-5.

18. McGinnis CS, Murrow LM, Gartner ZJ. DoubletFinder: Doublet Detection in Single-Cell RNA Sequencing Data Using Artificial Nearest Neighbors. *Cell Syst*. 2019; doi: 10.1016/j.cels.2019.03.003.

19. Domanska D, Majid U, Karlsen VT, Merok MA, Beitnes A-CR, Yaqub S, et al.. Single-cell transcriptomic analysis of human colonic macrophages reveals niche-specific subsets. *J Exp Med*. 2022; doi: 10.1084/jem.20211846.

20. La Manno G, Soldatov R, Zeisel A, Braun E, Hochgerner H, Petukhov V, et al.. RNA velocity of single cells. *Nature*. 2018; doi: 10.1038/s41586-018-0414-6.

21. Popescu D-M, Botting RA, Stephenson E, Green K, Webb S, Jardine L, et al.. Decoding human fetal liver haematopoiesis. *Nature*. 2019; doi: 10.1038/s41586-019-1652-y.

22. Xi NM, Li JJ. Benchmarking Computational Doublet-Detection Methods for Single-Cell RNA Sequencing Data. *Cell Syst*. 2021; doi: 10.1016/j.cels.2020.11.008.

23. Aran D, Looney AP, Liu L, Wu E, Fong V, Hsu A, et al.. Reference-based analysis of lung single-cell sequencing reveals a transitional profibrotic macrophage. *Nat Immunol*. 2019; doi: 10.1038/s41590-018-0276-y.

24. Wood DE, Lu J, Langmead B. Improved metagenomic analysis with Kraken 2. *Genome Biol*. 2019; doi: 10.1186/s13059-019-1891-0.

25. Jin S, Guerrero-Juarez CF, Zhang L, Chang I, Ramos R, Kuan C-H, et al.. Inference and analysis of cell-cell communication using CellChat. *Nat Commun*. 2021; doi: 10.1038/s41467-021-21246-9.

26. Yu G, Wang L-G, Han Y, He Q-Y. clusterProfiler: an R package for comparing biological themes among gene clusters. *OMICS*. 2012; doi: 10.1089/omi.2011.0118.

27. Cao J, Spielmann M, Qiu X, Huang X, Ibrahim DM, Hill AJ, et al.. The single-cell transcriptional landscape of mammalian organogenesis. *Nature*. 2019; doi: 10.1038/s41586-019-0969-x.

28. Mahmoudabadi G, Tabula Sapiens Consortium, Quake SR. Single Cell Transcriptomics Reveals the Hidden Microbiomes of Human Tissues. bioRxiv.

29. Galeano Niño JL, Wu H, LaCourse KD, Kempchinsky AG, Baryiames A, Barber B, et al.. Effect of the intratumoral microbiota on spatial and cellular heterogeneity in cancer. *Nature*. 2022; doi: 10.1038/s41586-022-05435-0.

30. Gardner PP, Paterson JM, McGimpsey S, Ashari-Ghomi F, Umu SU, Pawlik A, et al.. Sustained software development, not number of citations or journal choice, is indicative of accurate bioinformatic software. *Genome Biol*. 2022; doi: 10.1186/s13059-022-02625-x.

31. Hillje R, Pelicci PG, Luzi L. Cerebro: interactive visualization of scRNA-seq data. *Bioinformatics*. 2020; doi: 10.1093/bioinformatics/btz877.
