# Supplementary material for: Cellsnake: a user-friendly tool for single-cell RNA sequencing analysis
Source: Gigascience. 2023 Oct 27;12:giad091. doi: 10.1093/gigascience/giad091 (PMC10603768; doi:10.1093/gigascience/giad091)
Supplement: giad091_Response_to_Reviewer_Comments_Original_Submission [file giad091_response_to_reviewer_comments_original_submission.pdf]

Author's Response To Reviewer Comments

We thank the reviewers for their valuable comments. We addressed all the issues. For this review, we also updated the cellsnake to a new version NUMBER. We fixed bugs and improved performance. We rerun the submission version of cellsnake (v0.2.0.11) and updated the manuscript figures and repository files accordingly.

Reviewer reports:
Reviewer #1: The manuscript describes Cellsnake, a user-friendly tool for single-cell RNA sequencing analysis that targets non-expert users in the field of bioinformatics. Cellsnake operates as a command-line application, providing offline analysis capabilities for sensitive data. The integration of popular single-cell RNA-seq analysis software within Cellsnake, as described in Table 1, enhanced its utility as a comprehensive workflow. Cellsnake has different execution options (minimal, standard, and advanced) with varying outputs and execution times. The authors have provided well-structured online documentation, including helpful quick-start examples that facilitated easy understanding and usage of Cellsnake. The tool was tested using the Docker appliance and the provided fetal brain dataset and performed as expected. The manuscript explains the functions well, with the results reproduced from existing research using publicly available datasets.

The following issues need to be addressed by the authors.

1. The authors should include the citation for the Snakemake paper to acknowledge its contribution. https://doi.org/10.1093/bioinformatics/bts480

Thank you for the reviewer's comment. We have added the Snakemake citation. We have updated the information in the section "Cellsnake workflow and tools".

2. To support the claim of unique features in Cellsnake, a comparison with other similar methods, such as that on Galaxy (https://doi.org/10.1093/gigascience/giaa102), should be included.

Thank you for the reviewer's comment. We added to the manuscript a new table (Table 2) in the Discussion section comparing the main features of three holistic analysis tools: cellsnake, Cellenics and single-cell omics workbench (i.e.Galaxy workflow).

3. It is recommended to host the Docker container image on both the GitHub Container Registry and the Docker Hub for better availability and redundancy. The authors should publish the Dockerfile to enable users to build a container image, if needed.

Thank you for the reviewer's comment. Our Dockerfile is now available in our main repository (https://github.com/sinanugur/cellsnake/Dockerfile) and our workflow repository (https://github.com/sinanugur/scrna-workflow/Dockerfile). We added the publication version of Dockerfile to our Zenodo repository as well. Our Docker image is hosted at DockerHub (https://hub.docker.com/r/sinanugur/cellsnake).

4. Online documentation is missing a link to the fetal-liver example dataset (https://cellsnake.readthedocs.io/en/latest/fetalliver.html), which needs to be addressed. The fetal-brain dataset shared via Dropbox should also be deposited in the Zenodo repository to improve accessibility and long-term preservation.
Thank you for the reviewer's comment. We included the Zenodo link of the dataset. We also updated our online documentation to cover all features of Cellsnake including how fetal-liver samples were processed.

5. To assist users who want to use Cellsnake as a Snakemake workflow, the tool documentation should provide clear instructions on how to run Cellsnake as a single snakemake pipeline. This would be useful for

users who utilize existing workflow platforms to accept snakemake requests.
Thank you for the reviewer’s comment. We now updated the snakemake repository as well and put more comprehensive instructions (https://cellsnake.readthedocs.io/en/latest/). Cellsnake can be run as a tool or snakemake workflow as promised.

6. The benchmarking of Cellsnake must provide more precise specifications than simply referring to "a standard laptop" for computing requirements. My trial of "cellsnake integrated standard" with the fetal-brain dataset took more than 17 h via Docker execution on my M1 Max MacBook Pro. This may be because the provided Docker image is AMD-based, which let my MacBook run the container on a VM, but the recommended computational specifications will help users. The GitHub issue of the Cellsnake repository also mentioned that the software is not tested on Windows Conda, which should be mentioned at least in the online documentation.

Thanks for the reviewer's comprehensive testing and commenting on the issue.

Unfortunately it is not possible to run cellsnake natively on Apple Silicon (M1 or newer) for now. For example, Kraken2 is not compatible with this architecture and our Docker image is therefore AMD64-based. It is however possible to run without a Docker container using the Bioconda package. Yet still it has to be forced to use Osx64 architecture and all the dependencies can be resolved. This offers a faster experience than Docker container for Apple laptops.

We noted this explicitly in our online documentation. As soon as there is a native solution, we will recreate a new Docker image.

7. In the Data Availability section, please ensure that the correct formatting and consistent identifiers are used for public data, such as replacing SRP129388 with PRJNA429950 and E-MTAB-7407 with PRJEB34784, specifying that these IDs are from the Bioproject database. It is important to mention that EGA files are under controlled access, requiring user permission for retrieval.

Thank you for the reviewer’s comment. We updated accordingly.

8. The references in the manuscript need to be properly formatted to ensure the inclusion of publication years and DOIs where available.
Thank you for the reviewer’s comment. Now all the references have DOI numbers and publication years.

9. The help message from the Cellsnake command indicates that its default values are set for human samples. The authors should mention in the manuscript that the pipeline is configured for human samples and requires further configuration for use with samples from other organisms. A step-by-step guide to configuring the setting for the other species, including the reference data download, would be helpful in obtaining more audiences.

Thank you for the reviewer’s comment. We now put a few examples and config files for other species. Our updated documentation can be accessed here (https://cellsnake.readthedocs.io/en/latest/)

Reviewer #2: This paper offers an open-source tool, i.e., cellsnake, to perform single-cell data analysis. This cellsnake tool offers advanced features for standard users and facilitates downstream analyses in both R and Python environments. It is also designed for easy integration into existing workflows, allowing for rapid analyses of multiple samples. I like the incorporation design of the metagenome analysis in this tool, which makes it different with other available tools in single-cell analysis.

1) I looked through their tutorial, and have a specific question regarding the resolution parameter. I wonder if this resolution argument needs to be pre-selected? Or the cellsnake tool can automatically select a resolution parameter?
Thank you for the reviewer’s comment. The resolution parameter is quite important since it selects

granularity and many analyses on the downstream analyses mainly depend on how the clustering was done. Cellsnake can auto-select the resolution parameter and it works quite well if there are enough resources (memory and CPU). The user can also use the “clustree” plot to infer the optimum resolution parameter which is computationally less exhaustive.

2) Is it possible to add color legends in the umap? Rather than label all cell types on the umap. It can be very hard to distinguish the cell types, especially when there are many cell types available.
Thank you for the reviewer’s comment. This feature is already part of the cellsnake. In order to prepare the figures for the publication, we dropped legends. However, the legends are visible in our documentation and also in the supplementary files.

In large datasets, legends can cause overflow on the plots. We optimized the size of the plotting legends to prevent overflowing. Since the outputs are PDFs. The users can manually do some final work to create figure plots for their publication. We explore new ways to make cellsnake plots as easy as possible to use directly.

3) If the single-cell data is profiled from human tissue, is it also possible to use cellsnake to perform microbiome analysis?
Thank you for the reviewer’s comment. As long as the data is a single cell RNA dataset, microbiome analysis can be performed. It does not matter whether organisms or the tissue type. The only other requirement is the selection of the proper database to be able to use Kraken2.

4) I recommend the authors to compare cellsnake with other existing tools. Pros and cons need to be highlighted.
Thank you for the reviewer’s comment. As pointed out by the two reviewers, we included a table that compares the main features/functions of cellsnake with existing holistic tools/workflows. The table is added as table 3 in the discussion section.
