# Supplementary material for: Cellsnake: a user-friendly tool for single-cell RNA sequencing analysis
Source: Gigascience. 2023 Oct 27;12:giad091. doi: 10.1093/gigascience/giad091 (PMC10603768; doi:10.1093/gigascience/giad091)
Supplement: giad091_Response_to_Reviewer_Comments_Revision_1 [file giad091_response_to_reviewer_comments_revision_1.pdf]

Author's Response To Reviewer Comments

Dear Nicole,

Thanks for the fast processing from your end and also I would like thank GigaScience staff, very fast and efficient work.

I have finished all the corrections required and accepted your changes as intended. All references were checked and I dropped any hyperlinks inside the manuscript. I have registered cellsnake to the bio.tools and SciCrunch.org and both IDS were included.

We are planning to apply for additional funding for advancing Cellsnake. I hope we get the official acceptance so we can put this into our proposal.

Thanks for you help and consideration.

Cheers,
Sinan U. Umu, PhD
