# Supplementary material for: Cellsnake: a user-friendly tool for single-cell RNA sequencing analysis
Source: Gigascience. 2023 Oct 27;12:giad091. doi: 10.1093/gigascience/giad091 (PMC10603768; doi:10.1093/gigascience/giad091)
Supplement: giad091_Reviewer_1_Report_Original_Submission — Tazro Ohta -- 6/23/2023 Reviewed [file giad091_reviewer_1_report_original_submission.pdf]

Reviewer Report

Title: cellsnake: a user-friendly tool for single-cell RNA sequencing analysis

Version: Original Submission **Date:** 6/23/2023

Reviewer name: Tazro Ohta

Reviewer Comments to Author:

The manuscript describes Cellsnake, a user-friendly tool for single-cell RNA sequencing analysis that targets non-expert users in the field of bioinformatics. Cellsnake operates as a command-line application, providing offline analysis capabilities for sensitive data. The integration of popular single-cell RNA-seq analysis software within Cellsnake, as described in Table 1, enhanced its utility as a comprehensive workflow. Cellsnake has different execution options (minimal, standard, and advanced) with varying outputs and execution times. The authors have provided well-structured online documentation, including helpful quick-start examples that facilitated easy understanding and usage of Cellsnake. The tool was tested using the Docker appliance and the provided fetal brain dataset and performed as expected. The manuscript explains the functions well, with the results reproduced from existing research using publicly available datasets.
The following issues need to be addressed by the authors.
1. The authors should include the citation for the Snakemake paper to acknowledge its contribution. https://doi.org/10.1093/bioinformatics/bts480
2. To support the claim of unique features in Cellsnake, a comparison with other similar methods, such as that on Galaxy (https://doi.org/10.1093/gigascience/giaa102), should be included.
3. It is recommended to host the Docker container image on both the GitHub Container Registry and the Docker Hub for better availability and redundancy. The authors should publish the Dockerfile to enable users to build a container image, if needed.
4. Online documentation is missing a link to the fetal-liver example dataset (https://cellsnake.readthedocs.io/en/latest/fetalliver.html), which needs to be addressed. The fetal-brain dataset shared via Dropbox should also be deposited in the Zenodo repository to improve accessibility and long-term preservation.
5. To assist users who want to use Cellsnake as a Snakemake workflow, the tool documentation should provide clear instructions on how to run Cellsnake as a single snakemake pipeline. This would be useful for users who utilize existing workflow platforms to accept snakemake requests.
6. The benchmarking of Cellsnake must provide more precise specifications than simply referring to "a standard laptop" for computing requirements. My trial of "cellsnake integrated standard" with the fetal-brain dataset took more than 17 h via Docker execution on my M1 Max MacBook Pro. This may be because the provided Docker image is AMD-based, which let my MacBook run the container on a VM, but the recommended computational specifications will help users. The GitHub issue of the Cellsnake repository also mentioned that the software is not tested on Windows Conda, which should be mentioned at least in the online documentation.
7. In the Data Availability section, please ensure that the correct formatting and consistent identifiers

are used for public data, such as replacing SRP129388 with PRJNA429950 and E-MTAB-7407 with PRJEB34784, specifying that these IDs are from the Bioproject database. It is important to mention that EGA files are under controlled access, requiring user permission for retrieval.
8. The references in the manuscript need to be properly formatted to ensure the inclusion of publication years and DOIs where available.
9. The help message from the Cellsnake command indicates that its default values are set for human samples. The authors should mention in the manuscript that the pipeline is configured for human samples and requires further configuration for use with samples from other organisms. A step-by-step guide to configuring the setting for the other species, including the reference data download, would be helpful in obtaining more audiences.

Methods

Are the methods appropriate to the aims of the study, are they well described, and are necessary controls included? Choose an item.

Conclusions

Are the conclusions adequately supported by the data shown? Choose an item.

Reporting Standards

Does the manuscript adhere to the journal's guidelines on minimum standards of reporting? Choose an item.

Choose an item.

Statistics

Are you able to assess all statistics in the manuscript, including the appropriateness of statistical tests used? Choose an item.

Quality of Written English

Please indicate the quality of language in the manuscript: Choose an item.

Declaration of Competing Interests

Please complete a declaration of competing interests, considering the following questions:

- Have you in the past five years received reimbursements, fees, funding, or salary from an organisation that may in any way gain or lose financially from the publication of this manuscript, either now or in the future?

- Do you hold any stocks or shares in an organisation that may in any way gain or lose financially from the publication of this manuscript, either now or in the future?
- Do you hold or are you currently applying for any patents relating to the content of the manuscript?
- Have you received reimbursements, fees, funding, or salary from an organization that holds or has applied for patents relating to the content of the manuscript?
- Do you have any other financial competing interests?
- Do you have any non-financial competing interests in relation to this paper?

If you can answer no to all of the above, write 'I declare that I have no competing interests' below. If your reply is yes to any, please give details below.

I declare that I have no competing interests

I agree to the open peer review policy of the journal. I understand that my name will be included on my report to the authors and, if the manuscript is accepted for publication, my named report including any attachments I upload will be posted on the website along with the authors' responses. I agree for my report to be made available under an Open Access Creative Commons CC-BY license (http://creativecommons.org/licenses/by/4.0/). I understand that any comments which I do not wish to be included in my named report can be included as confidential comments to the editors, which will not be published.

Choose an item.

To further support our reviewers, we have joined with Publons, where you can gain additional credit to further highlight your hard work (see: https://publons.com/journal/530/gigascience). On publication of this paper, your review will be automatically added to Publons, you can then choose whether or not to claim your Publons credit. I understand this statement.

Yes Choose an item.
