# Supplementary material for: Cellsnake: a user-friendly tool for single-cell RNA sequencing analysis
Source: Gigascience. 2023 Oct 27;12:giad091. doi: 10.1093/gigascience/giad091 (PMC10603768; doi:10.1093/gigascience/giad091)
Supplement: giad091_Reviewer_2_Report_Original_Submission — Qianqian Song -- 7/3/2023 Reviewed [file giad091_reviewer_2_report_original_submission.pdf]

Reviewer Report

Title: cellsnake: a user-friendly tool for single-cell RNA sequencing analysis

Version: Original Submission **Date:** 7/3/2023

Reviewer name: Qianqian Song

Reviewer Comments to Author:

This paper offers an open-source tool, i.e., cellsnake, to perform single-cell data analysis. This cellsnake tool offers advanced features for standard users and facilitates downstream analyses in both R and Python environments. It is also designed for easy integration into existing workflows, allowing for rapid analyses of multiple samples. I like the incorporation design of the metagenome analysis in this tool, which makes it different with other available tools in single-cell analysis.
1) I looked through their tutorial, and have a specific question regarding the resolution parameter. I wonder if this resolution argument needs to be pre-selected? Or the cellsnake tool can automatically select a resolution parameter?
2) Is it possible to add color legends in the umap? Rather than label all cell types on the umap. It can be very hard to distinguish the cell types, especially when there are many cell types available.
3) If the single-cell data is profiled from human tissue, is it also possible to use cellsnake to perform microbiome analysis?
4) I recommend the authors to compare cellsnake with other existing tools. Pros and cons need to be highlighted.

Methods

Are the methods appropriate to the aims of the study, are they well described, and are necessary controls included? Choose an item.

Conclusions

Are the conclusions adequately supported by the data shown? Choose an item.

Reporting Standards

Does the manuscript adhere to the journal's guidelines on minimum standards of reporting? Choose an item.

Choose an item.

Statistics

Are you able to assess all statistics in the manuscript, including the appropriateness of statistical tests used? Choose an item.

Quality of Written English

Please indicate the quality of language in the manuscript: Choose an item.

Declaration of Competing Interests

Please complete a declaration of competing interests, considering the following questions:

- Have you in the past five years received reimbursements, fees, funding, or salary from an organisation that may in any way gain or lose financially from the publication of this manuscript, either now or in the future?
- Do you hold any stocks or shares in an organisation that may in any way gain or lose financially from the publication of this manuscript, either now or in the future?
- Do you hold or are you currently applying for any patents relating to the content of the manuscript?
- Have you received reimbursements, fees, funding, or salary from an organization that holds or has applied for patents relating to the content of the manuscript?
- Do you have any other financial competing interests?
- Do you have any non-financial competing interests in relation to this paper?

If you can answer no to all of the above, write 'I declare that I have no competing interests' below. If your reply is yes to any, please give details below.

I declare that I have no competing interests.

I agree to the open peer review policy of the journal. I understand that my name will be included on my report to the authors and, if the manuscript is accepted for publication, my named report including any attachments I upload will be posted on the website along with the authors' responses. I agree for my report to be made available under an Open Access Creative Commons CC-BY license (http://creativecommons.org/licenses/by/4.0/). I understand that any comments which I do not wish to be included in my named report can be included as confidential comments to the editors, which will not be published.

Choose an item.

To further support our reviewers, we have joined with Publons, where you can gain additional credit to further highlight your hard work (see: https://publons.com/journal/530/gigascience). On publication of this paper, your review will be automatically added to Publons, you can then choose whether or not to claim your Publons credit. I understand this statement.

Yes Choose an item.
